# Quality of gout care in the emergency departments: a multicentre study

**DOI:** 10.1186/s12873-020-00319-w

**Published:** 2020-04-20

**Authors:** Patapong Towiwat, Pariwat Phungoen, Kitti Tantrawiwat, Pavita Laohakul, Duangkamol Aiewruengsurat, Chokchai Thanadetsuntorn, Nopparat Ruchakorn, Passagorn Sangsawangchot, Bodin Buttham

**Affiliations:** 1 Department of Internal Medicine, Faculty of Medicine, Naresuan University, Phitsanulok, 65000 Thailand; 2 Department of Emergency Medicine, Faculty of Medicine, Khon Kaen University, Khon Kaen, Thailand; 3 Department of Surgery, Faculty of Medicine, Naresuan University, Phitsanulok, Thailand; 4 Department of Emergency Medicine, Faculty of Medicine, Chiang Mai University, Chiang Mai, Thailand; 5 Allergy and Rheumatology Unit, Division of Internal Medicine, Faculty of Medicine, Prince of Songkla University, Songkla, Thailand; 6 Division of Allergy Immunology and Rheumatology, Department of Internal Medicine, Faculty of Medicine, Ramathibodi Hospital, Mahidol University, Bangkok, Thailand; 7 Department of Internal Medicine, Faculty of Medicine, Srinakharinthawirot University, Bangkok, Thailand

**Keywords:** Gout, Management, Quality of care, Emergency department

## Abstract

**Background:**

To report on prevalence of gout flare in emergency departments and to report the quality of gout care in emergency departments and causes of admission at emergency departments.

**Methods:**

A retrospective chart review of visits that had a primary diagnosis in gout by the International Classification of Diseases, the tenth revision, at emergency departments from 6 universities in Thailand over a 5 year period from 1 January 2012 to 31 December 2016.

**Results:**

Six hundred thirty-two visits were included to the study. Prevalence of gout flare in emergency departments was 0.04. Only 29.3% of the visits had arthrocentesis. 628/632 (99.4%) and 519/585 (88.7%) of the visits were prescribed medications in emergency departments and had home medications, respectively. Although all visits that were prescribed colchicine in emergency departments received adequate doses of colchicine, it was also found that more than 2.4 mg/day of colchicine was prescribed (3/394, 0.8%) for home medications. In addition, 183/343 (53.4%) of the visits with normal renal function were prescribed non-steroidal anti-inflammatory drugs (NSAIDs). However, prescribed NSAIDs in abnormal renal function (42/343, 12.2%) was also found. The interruption of dosing, including increase, decrease, addition or discontinuance of urate lowing therapy in a gout flare period was 42/632 (6.6%). The most common cause of admission was acute gouty arthritis (31/47, 66.0%).

**Conclusions:**

Quality of gout care in the emergency departments was not good. Inappropriate management of gout flare in emergency departments was demonstrated in our study, particularly with regard to investigations and pharmacological management. Gaps between clinicians and guidelines, the knowledge of clinicians, and overcrowding in emergency departments were hypothesized in the results.

## Background

Gout flare (GF) that was a characteristic of intense inflammatory arthritis was common among gouty patients [1, 2]. The different managements between GF and asymptomatic gout or post inflammation subside were investigations and medications, particularly dosage and timing of colchicine, route of administration, duration and dosage of corticosteroids, and adjusted dosage of allopurinol [3–5]. The severe painful GF was the major clinical burden of gout [6]. This symptom needed urgent medical treatment to stop severe pain and inflammation as soon as possible and caused a visit to the emergency departments (EDs) [7].

Although the national guideline of gout was published [3, 5], suboptimal management has been found in EDs, out-patient departments, and admitted patients [8–14]. The common pitfalls involving inappropriate management of GF in hospitals were knowledge, investigations and medications [10, 11, 14, 15]. Non-compliance with the guideline in gout and other diseases included perceptions of illness by clinicians, unawareness, factors of patients, and appropriation of guidelines in real practice [16–19].

Because of overcrowding in EDs from increased patient volume [20], missed diagnoses, inappropriate order and interpreted diagnostic testing, poor adherence to guideline-recommended treatment, and long emergency department length of stay were found [21, 22]. In addition, high workload and knowledge of staff were problems in EDs, affecting patients safety and poor outcome [20, 23, 24]. GF was a cause of arthritis that was found in EDs and had been increasing [10, 25, 26]. However, suboptimal care of GF patients in EDs was reported [7, 10, 27, 28]. Moreover, some gouty patients who visited EDs were also admitted to hospital [29].

To date there have been no studies of the prevalence of GF and quality of gout care in EDs in university hospitals based on a national guideline. Therefore, following the 2012 Thai Rheumatism Association Guidelines for Management of Gout (TRA-GMG), the objectives of this study were to report on prevalence of GF in EDs, the quality of gout care, medications used, investigations, length of stay at EDs, causes of admission, and follow up period after discharge in EDs of university hospitals in Thailand.

## Methods

### Patient selection

The site of the study was the EDs. Data of visits to EDs with gout from 1 January 2012 to 31 December 2016 were reviewed at discharge from 6 university EDs in Thailand, including Naresuan University Hospital (Naresuan University), Maharaj Nakorn Chiang Mai Hospital (Chiang Mai University), Songklanagarind Hospital (Prince of Songkla University), Srinagarind Hospital (Khon Kaen University), Ramathibodi Hospital (Mahidol University), and HRH Princess Maha Chakri Sirindhorn Medical Center (Srinakharinthawirot University). Ethical approval was received from each center. Permission to review and use the data of these patients’ records was obtained from the Dean or the director of each centre.

For this study, we limited analyses of visits to those where patients were diagnosed with gout as the primary diagnosis at ED visits. We identified only the primary diagnosis of gout, using the International Classification of Diseases, the tenth revision (ICD-10) code, including M10.0, M10.1, M10.2, M10.3, M10.4 and M10.9 at EDs when the patients were discharged. All medical records were reviewed. If visits had data missing of medications in EDs or home medications (HM), these visits were excluded.

### Variable definition

Definite gouty arthritis was the presence of monosodium urate crystals (MSU) in synovial fluid as performed in the EDs or at least a score of 8 according to the 2015 American College of Rheumatology (ACR)/European League Against Rheumatism (EULAR) classification for gout [30]. Variables included sex; age; blood pressure; underlining diseases; medications given during the ED visit and those given as a prescription, including colchicine, non-steroidal anti-inflammatory drugs (NSAIDs) that included cyclooxygenase (COX)-1 and COX-2 inhibitors, steroids, proton pump inhibitor (PPI) and others; consultations requested; number of joints involved; attack duration; presence of tophi; diagnostic tests, including joint aspirations, x-rays, and blood tests within the last 3 months and serum uric acid (SUA); duration of medication prescribed after discharge; and others. Defined abnormal renal function, including estimated glomerular filtration rate (eGFR) within the last 3 month or recently in EDs was of less than 60 ml/min/1.73m^2^ [31]. If some patients had a history of gout, the dosage of medications of urate lowering therapy (ULT), including uricostatic and uricosuric drugs that were recorded in the latest visit was reviewed. Also, the length of stay at the EDs was calculated. Causes of admission and follow up period after discharge from the EDs were also reviewed.

Acute arthritis was defined as the presence of acute painful, swollen, and tender joints [32] within 2 weeks. GF was defined as a clinical presentation of acute arthritis in visits that had been identified by the primary diagnosis of gout (as mentioned above). Early and late treatment referred to treatment received within 72 or more than 72 h after onset of GF, respectively.

Monotherapy was a medication that was chosen for treatment, including colchicine, NSAIDs, and corticosteroids [3]. Combination therapy was more than 1 medication prescription. Indication for PPI use in patients prescribed NSAIDs was evaluated following the guidelines for prevention of NSAID-related ulcer complications [33]. Severe liver disease was defined as history of liver cirrhosis, cholangiocarcinoma, or any metastatic liver cancer. Abnormal renal function and severe liver disease were contraindications of NSAIDs for management in GF.

The pitfalls in management of GF in this present study were divided into 2 categories including investigations and managements. For investigation, *over investigation* was defined as investigations which included SUA *or* plain film of the inflammatory joint(s) that were investigated at EDs, whereas *under investigation* was defined as no evaluation of renal function within the last 3 months or recently in EDs *or* arthrocentesis was not performed at EDs. For management, *over treatment* was defined as combination therapies (NSAIDs plus corticosteroids *or* triple regimen (NSAIDs and colchicine plus corticosteroids)) *or* prescribing of PPI or gastro-protective agent in visits without indications [33] (only in HM). *Under treatment* was defined as no pharmacological prescription for management of GF in EDs or HM *or* no prescribed PPI or gastro-protective agent in visits that had indications (only in HM) [33]. *Overuse medication* was defined as prescribing colchicine in late treatment of GF *or* prescription of more than 4 Tabs (2.4 mg/day) of colchicine in normal kidney function *or* prescribing of NSAIDs in abnormal renal function or sever liver disease *or* inadequate dosage of corticosteroids *or* administration of balm.

### Quality measures

Our study followed the 2012 TRA-GMG [4] in measuring gout care quality in EDs. In this study, we aimed to determine the quality of gout care at EDs in 3 main areas: 1) the diagnosis of gout, 2) education or non-pharmacological of acute gout care, and 3) investigations and management of acute gout care.

### Statistics

Descriptive and summary statistics were performed. Continuous variables were described as ±SD, and categorical variables were described as percentage. Comparison between the 2 groups of continuous variables and categorical variables was performed using the Student’s t-test and Chi-square or Fisher’s exact test, where appropriate. Data were analysed by subgroup analysis between definite gout and diagnosis by ICD-10. A *p*-value of less than 0.05 was considered a statistically significant difference.

## Result

Visits in EDs totalled 1,621,403. Of these, 1908 visits were identified from ICD10, however, medical records were missing or the diagnosis of gout could not be identified in the medical record after review in 1276 visits. These visits were excluded from the study. The flow of visits in this study was shown in Additional file 1. Therefore, 632 with the diagnosis of gout by ICD-10 were included in this study.

Hence, prevalence of GF in EDs was 0.04. M10.0 was the most common ICD-10 gout diagnosis (361, 57.1%). The mean age was 57.29 (±16.90) years old. Monoarthritis (476, 75.3%) was the most common form of presentation. The most commonly involved joint (294, 46.5%) was the ankle joint. The baseline demographic and clinical characteristics of data were summarized in Table 1.

**Table 1:** Baseline demographic and clinical characteristics

Variable	Total N (%) (*n* = 632)	Definite gout^a^ N (%) (*n* = 266)	Diagnosis by ICD-10 N (%) (*n* = 366)	*P*-value*
Male	521 (82.4)	211 (79.3)	310 (84.7)	0.08
Age, years, mean (±SD)	57.29 (±16.90)	58.77 (±17.96)	56.21 (±16.03)	0.060
Temperature ≥ 37.8 °c	89 (14.1)	55 (20.7)	34 (9.3)	< 0.001
Blood pressure ≥ 140/90 mmHg	147 (23.3)	55 (20.7)	92 (25.1)	0.308
History of gout	427 (67.6)	174 (65.4)	253 (69.1)	0.325
Shift				0.002
- 08.01 AM - 04.00 PM	265 (41.9)	131 (49.3)	134 (36.6)	
- 04.01 PM - 00.00 AM	247 (39.1)	98 (36.8)	149 (40.7)	
- 00.01 AM - 08.00 AM	120 (19.0)	37 (13.9)	83 (22.7)	
Number of joints involved				0.153
- Monoarthritis	476 (75.3)	190 (71.4)	286 (78.1)	
- Oligoarthritis	126 (19.9)	61 (23.0)	65 (17.8)	
- Polyarthritis	30 (4.8)	15 (5.6)	15 (4.1)	
Identity of joint(s) involved				
- Knees	215 (34.0)	119 (44.7)	96 (26.2)	< 0.001
- Ankles	294 (46.5)	124 (46.6)	170 (46.5)	0.967
- The first metatarsophalangeal joint	153 (24.2)	51 (19.2)	102 (27.9)	0.012

*p value was compared between definite gout and diagnosis by ICD-10

^a^Definite diagnosis of gout was made if MSU crystals in synovial fluid as identified in the EDs or at least a score of 8 according the ACR/EULAR classification criteria

Arthrocentesis was performed in 185 (29.3%) of all visits, in which the knee joint was performed in 117 (63.2%) of the visits. Overall, 585 (92.6%) and 47 (7.4%) of all visits were discharged home and admitted to the hospitals, respectively. Of the total visits, 157 (24.8%) were consulted to the internal medicine or orthopaedics department. The average length of stay at EDs was 2.16 h (±2.16 h).

Following the 2012 TRA-GMG, three main items including 1) the diagnosis of gout, 2) education or non-pharmacological acute gout care, and 3) investigations and management of acute gout care were evaluated.

A: Diagnosis

*The definite gout diagnosis was evaluated.*

Of 185 visits that arthrocentesis were applied, MSU was identified in 158/185 (85.4%) of the visits. Of the total visits for which a definite gout diagnosis was made if MSU crystals were identified or the visit had a score of at least 8 according to the 2015 ACR/EULAR classification criteria for gout, there were 266 (42.1%) of the total visits.

B: Education or non-pharmacological therapy

*Education of acute gout care or non-pharmacological therapy, including education on diet, alcohol avoidance, rest for involved joint or no massage, use of ice pack, and elevation of the involved joint was assessed.*

Four hundred patients (63.3%) of the total visits were educated regarding diet, alcohol avoidance, rest of involved joint or no massage, use of ice pack, and elevation of involved joint.

C: Investigations and management

For investigations at EDs, the results were shown in Table 2. Six hundred twenty-eight visits (99.4%) of the total visits were prescribed medications for acute gout care in EDs. Most of the visits also had HM (Table 2). Medications for visits which were prescribed in EDs and/or had HM, monotherapy and combination therapy, were shown in Table 2.

1. *Colchicine*

1.1 *Colchicine was administered in early treatment of GF that occurred within 72 h after GF onset.*

**Table 2:** Investigations and regimens of pharmacological therapy for management of GF at EDs

Items	Acute management in EDs N (%) (*N* = 632)	Home medications N (%) (*N* = 585)
Investigations		
- Complete blood count	265 (41.9)	NA
- Renal function test	302 (47.8)	NA
- Serum uric acid level	219 (34.7)	NA
- Radiographic examination (plain film)	144 (22.8)	NA
Prescribed pharmacological therapies for management of GF		
- Yes	628 (99.4)	519 (88.7)
- No	4 (0.6)	66 (11.3)
Monotherapy	(*n* = 628)	(*n* = 519)
- Colchicine	167 (26.6)	186 (35.8)
- NSAIDs	95 (15.1)	76 (14.6)
- Corticosteroids	51 (8.1)	40 (7.7)
- Other analgesics or opiate analgesic	42 (6.7)	5 (1.0)
Combination therapy	(*n* = 628)	(*n* = 519)
- NSAIDs plus colchicine	233 (37.1)	193 (37.2)
- NSAIDs plus corticosteroids	10 (1.6)	4 (0.8)
- Colchicine plus corticosteroids	25 (4.0)	12 (2.3)
- NSAIDs and colchicine plus corticosteroids	5 (0.8)	3 (0.6)

*NSAIDs* Non-steroidal anti-inflammatory drugs, *GF* Gout flare

Respectively, at onset of treatment, 372/628 (59.3%) and 340/519 (65.5%) visits with GF were prescribed colchicine as monotherapy or combination therapy in early treatment at EDs and for HM, respectively (Table 3).

1.2 *Actually, there is no definite dosage for colchicine in acute gout treatment. According to TRA-GMG, the dosage should not be more than 4 tabs (2.4 mg/day) in normal kidney function. There was a guideline that suggests reducing the dosage of colchicine if creatinine clearance < 30 mL/min. However, the TRA-GMG does not mention this. However, currently, there is no recommendation for the use of high dose colchicine in acute gout (colchicine 0.6 mg every 2 h until arthritis improved).*

**Table 3:** Pharmacological therapies used in management of GF in the EDs following the 2012 TRA-GMG

Items/Pharmacological therapy	Acute management in EDs N (%) (*n* = 632)	Home medications N (%) (*n* = 585)
Prescribed medications for management of GF	628 (99.4)	519 (88.7)
Colchicine		
Prescribed pharmacological therapies for management of GF	(*n* = 628)	(*n* = 519)
- Early treatment^a^		
- Colchicine	372 (59.3)	340 (65.5)
- Other	169 (26.9)	106 (20.4)
- Late treatment^a^		
- Colchicine	58 (9.2)	54 (10.4)
- Other	29 (4.6)	19 (3.7)
The dosage of colchicine^c^	(*n* = 430)	(*n* = 394)
- ≤4 tabs (≤2.4 mg/day)	430 (100)	391 (99.2)
- > 4 tabs (> 2.4 mg/day)	0 (0)	3 (0.8)
Non-steroidal anti-inflammatory drugs		
Prescribed pharmacological therapies for management of GF	(*n* = 628)	(*n* = 519)
- Early treatment^a^		
- NSAIDs	300 (47.8)	242 (46.6)
- Other	241 (38.4)	204 (39.3)
- Late treatment^a^		
- NSAIDs	43 (6.8)	34 (6.6)
- Other	44 (7.0)	39 (7.5)
NSAIDs^d^ and renal function	(*n* = 343)	(*n* = 276)
- Normal renal function (eGFR ≥60 ml/min/1.73m^2^)	183 (53.4)	151 (54.7)
- Abnormal renal function (eGFR < 60 ml/min/1.73m^2^)	42 (12.2)	30 (10.9)
- Unevaluated renal function	118 (34.4)	95 (34.4)
NSAIDs^d^ and severe liver disease^b^	(*n* = 343)	(*n* = 276)
- No severe liver disease	342 (99.7)	275 (99.6)
- Severe liver disease	1 (0.3)	1 (0.4)
Adequate duration (approximate 7 days) of NSAIDs^d^		(*n* = 276)
- Yes	–	117 (42.4)
- No	–	159 (57.6)
NSAIDs^d^ and PPI or gastro-protective agent use		(*n* = 276)
- Adequate	–	184 (66.7)
- Inadequate		
- Over use	–	40 (14.5)
- Under use	–	52 (18.8)
Corticosteroid		
Adequate dose (prednisolone 0.5–1 mg/kg/day (or equivalent)) of corticosteroid^e^		(*n* = 59)
- Yes	–	38 (64.4)
- No	–	21 (35.6)
The adequate duration (7–10 days) of corticosteroid^e^		(*n* = 59)
- Yes	–	16 (27.1)
- No	–	43 (72.9)

*EDs* Emergency departments, *NSAIDs* Non-steroidal anti-inflammatory drugs, *GF* Gout flare, *eGFR* Estimated glomerular filtration rate, *PPI* Proton pump inhibitor

^a^Early and late treatment of gout flare that was less than or equal to 72 and was more than 72 h after attack onset, respectively

^b^Severe liver disease defined as history of liver cirrhosis, cholangiocarcinoma, or any metastatic liver cancer

^c^Irrespective of onset treatment of GF, the number of patients prescribed colchicine, referred to as monotherapy and combination therapy at EDs: monotherapy (*n* = 167), NSAIDs plus colchicine (*n* = 233), colchicine plus corticosteroids (*n* = 25), NSAIDs and colchicine plus corticosteroids (*n* = 5) and monotherapy and combination therapy as HM: monotherapy (*n* = 186), NSAIDs plus colchicine (*n* = 193), colchicine plus corticosteroids (*n* = 12), NSAIDs and colchicine plus corticosteroids (*n* = 3).

^d^Irrespective of onset treatment of GF, the number of patients that were prescribed NSAIDs referred to as monotherapy and combination therapy at EDs: monotherapy (*n* = 95), NSAIDs plus colchicine (*n* = 233), NSAIDs plus corticosteroids (*n* = 10), NSAIDs and colchicine plus corticosteroids (*n* = 5) and monotherapy and combination therapy at HM: monotherapy (*n* = 76), NSAIDs plus colchicine (*n* = 193), NSAIDs plus corticosteroids (*n* = 4), NSAIDs and colchicine plus corticosteroids (*n* = 3).

^e^Irrespective of onset treatment of GF, the number of patients that were prescribed corticosteroid referred to as monotherapy and combination therapy at HM: monotherapy (*n* = 40), NSAIDs plus corticosteroid (*n* = 4), colchicine plus corticosteroids (*n* = 12), NSAIDs and colchicine plus corticosteroids (*n* = 3)

Although all visits that were administered monotherapy or a combination therapy of colchicine in EDs were prescribed an adequate dose of colchicine, colchicine that was prescribed for more than 4 tabs/day was presented for HM (Table 3).

2. *Non-steroidal anti-inflammatory drugs*

NSAIDs including monotherapy or combination therapy were prescribed at EDs and for HM (Table 2). Of the 343 visits that were prescribed NSAIDs as monotherapy and combination therapy in EDs, intramuscular injection and intravascular route were administered in 174/343 (50.7%) and 12/343 (3.5%) of visits, respectively.

2.1 *NSAIDs can be used in early or late treatment of GF (either within or after 72 h of acute onset). NSAIDs should not be used in acute heart failure, severe liver disease, and abnormal renal function. For GF, the duration of NSAID therapy is usually approximate 7 days. Other than that, multiple NSAIDs use or only aspirin use is not recommended.*

Respectively, at onset of treatment, NSAIDs treatment, both monotherapy and combination therapy, was presented in Table 3. About one half of visits that were administered NSAIDs were prescribed in normal renal function in acute management in EDs and for HM (Table 3).

NSAIDs were also prescribed in abnormal renal function or severe liver disease at EDs and for HM (Table 3). Of 343 visits that were prescribed NSAIDs in EDs, multiple NSAID use was found for 1/343 (0.3%) of the visits. The average duration of NSAID therapy was 7.4 (±3.6) days.

2.2 *The oral route is preferred. However, if patients cannot take NSAIDs by mouth, then parenteral therapy can be used*.

NSAIDs were administrated at EDs by intramuscular injection and intravascular route (as mentioned above). However, the oral route of NSAIDs was the preferred prescription in these visits.

2.3 *Adequate and optimal PPI or gastro-protective agent should be combined with NSAIDs (other than the coxibs group) for patients who are at risk for gastrointestinal bleeding.*

For HM, of the 276 visits that were prescribed NSAIDs, including monotherapy or combination therapy, 184/276 (66.7%) visits had adequate and optimal for PPI or gastro-protective agents (Table 3).

Despite having no indication for PPI or gastro-protective agent with NSAIDs, 40/276 (14.5%) of the visits were still administered PPI or gastro-protective agents.

3. *Corticosteroid*

3.1 *Corticosteroids will be recommended only in patients who have a contraindication for colchicine or NSAIDs. These include impaired renal function, gastrointestinal bleeding, severe liver disease, or no response to NSAIDs.*

Of the 200 and 150 visits that had a contraindication for colchicine or NSAIDs in EDs and for HM, respectively, monotherapy of systemic corticosteroid was prescribed in 42/200 (21.0%) and 35/150 (23.3%) of the visits in EDs and for HM, respectively.

3.2 *The adequate dose of corticosteroids is prednisolone 0.5–1 mg/kg/day (or equivalent) for the duration of 7–10 days. For monoarthritis, intra-articular corticosteroid injection can be considered.*

Out of 91 visits prescribed corticosteroid, including monotherapy or combination therapy in EDs, intra-articular corticosteroid injection and intravascular form (dexamethasone) were administered in 5/91 (5.5%) and 28/91 (30.8%) of visits, respectively.

In accordance with the 2012 TRA-GMG, the adequate dose and adequate duration of corticosteroids in HM was shown in Table 3. The average duration of corticosteroids was 6.4 (±3) days.

4. *Other analgesic or opiate analgesic can be used in combination with colchicine and NSAIDs*

Of the 495 and 455 visits that were administered monotherapy of colchicine and NSAIDs or combination therapy between colchicine and NSAIDs in EDs and for HM, respectively, 42/495 (8.5%) and 66/455 (14.5%) of the visits were also given an analgesic in EDs and for HM, respectively. The common analgesic that was combined was tramadol in both EDs (27/42, 64.3%) and for HM (27/66, 40.9%).

5. *For the patients who received ULT and develop GF, these medications should not be interrupted or stopped. In addition, in patients who do not receive ULT, these drugs also should not be initiated during GF.*

Interruption of dosing, including increase, decrease, addition, or discontinuance in the GF period was found for 42 (6.6%) of all visits in EDs. Among the 42 visits that had an interruption in dosing of ULT, an increase in the dose of ULT (24/42, 57.1%) was the most common.

6. *All patients who have improved after GF should have an appointment to follow up for evaluation of the risks or factors that can affect SUA and to evaluate complications of gout disease.*

Most visits (436, 69.0%) had an appointment to follow up. The average follow up period was 9.6 (±8.3) days.

Apart of the 2012 TRA-GMG, of 519 visits that had medications for management in HM, balm was administered to 6/519 (1.2%) of the visits. Although combination therapy was not mentioned in the 2012 TRA-GMG, these therapies were presented (Table 2). Moreover, other analgesics or opiate analgesics that were the only mono-therapy were presented in Table 2. The pitfalls of management of GF in EDs including investigations and managements were presented in Table 4.

**Table 4:** The pitfalls of management of GF in EDs

Pitfalls of management	Acute management in EDs N (%) (*n* = 632)	Home medications N (%) (*n* = 585)
Investigations		
*- Over investigation*	264 (41.8)	–
*- Under investigation*	493 (78.0)	–
Managements		
*- Over treatment*	15 (2.4)	47 (8.0)
*- Under treatment*	4 (0.6)	118 (20.2)
*- Overuse medication*	99 (15.7)	83 (14.2)

As inclusion criteria, this study selected the primary diagnosis of gout using the ICD-10 code when the patients were discharged at EDs. However, the results that showed statistical significance between a definite gout and ICD-10 diagnosis were shown in Table 5.

**Table 5:** Significant items/recommendations between a definite gout and diagnosis only by ICD-10

Items/recommendations	Total N (%) (*n* = 632)	Definite gout^a^ N (%) (*n* = 266)	ICD-10 N (%) (*n* = 366)
Investigations in EDs			
*-* Complete blood count**	265 (41.9)	152 (57.1)	113 (30.9)
*-* Renal function test**	302 (47.8)	161 (60.5)	141 (38.5)
Arthrocentesis**	185 (29.3)	161 (60.5)	24 (6.6)
Consultation**	157 (24.8)	105 (39.5)	52 (14.2)
Average time in EDs**, hour, mean (±SD)	2.16 (2.16) (*n* = 369)	3.05 (2.72) (*n* = 162)	1.46 (1.19) (*n* = 207)
Monotherapy of colchicine			
*-* In EDs*	167/628 (26.6)	93/265 (35.1)	74/363 (20.4)
*-* In HM*	186/519 (35.8)	91/204 (44.6)	95/315 (30.2)
NSAIDs^b^ can be used in early or late treatment of GF.			
*-* In EDs*	343/628 (54.6)	121/265 (45.7)	222/363 (61.2)
NSAIDs^b^ should be used in normal renal function.			
*-* In EDs*	183/343 (53.4)	85/121 (70.5)	98/222 (44.1)
NSAIDs^b^ were used in unevaluated renal function.			
*-* In EDs**	118/343 (34.4)	21/121 (17.4)	97/222 (43.7)
*-* In HM*	95/276 (34.4)	17/96 (17.7)	78/180 (43.3)
All patients who have improved after acute GF should have an appointment to follow up for evaluation of the risks or factors that can affect SUA and to evaluate complication of gout disease*.	436/632 (69.0)	216/266 (81.2)	220/366 (60.1)

*EDs* Emergency departments, *HM* home medications, *NSAIDs* Non-steroidal anti-inflammatory drugs, *GF* Gout flare, *SUA* Serum uric acid

*p* value was compared between definite gout and ICD-10 group

* *p* value< 0.05, ** *p* value< 0.001

^a^Definite diagnosis of gout was made if MSU crystals in synovial fluid as identified in the EDs or at least a score of 8 according the ACR/EULAR classification criteria.

^b^Including monotherapy and combination therapy.

For the cause of admission in visits that had GF, of 47 visits that were admitted in hospitals, acute gouty arthritis that was the cause of admission for 6/47 (12.8%) of the visits. However, 25/47 (53.2%) of the visits were admitted for clinical observation of septic arthritis. Finally, these visits were discharged and diagnosed as acute gouty arthritis. The final causes of admission that included other conditions as well as GF at EDs were shown in Table 6. Overall, gout was the main cause of admission (66.0%).

**Table 6:** The final causes of admission included conditions as well as GF at EDs

The final causes of admission	Total N (%), (*n* = 47)
Acute gouty arthritis	31 (66.0)
Acute kidney injury	3 (6.4)
Respiratory tract infection	2 (4.3)
Upper gastrointestinal hemorrhage	2 (4.3)
Cerebrovascular disease (stroke)	1 (2.1)
Congestive heart failure	1 (2.1)
Hypertensive urgency	1 (2.1)
Electrolyte imbalance	1 (2.1)
Urinary tract infection	1 (2.1)
Infected tophi	2 (4.3)
Infected CAPD	1 (2.1)

*CAPD* Continuous Ambulatory Peritoneal Dialysis

## Discussion

Prevalence of GF in EDs was 0.04. Although most visits had management in EDs and for HM, based on the 2012 TRA-GMG, there was inappropriate management of GF in EDs demonstrated in this study. Only the prescription dose of colchicine in EDs, but not in HM, was according to the 2012 TRA-GMG. Acute gouty arthritis was the most common cause of admission among visits.

Prevalence of GF in EDs in this study was less than in previous studies [10, 25]. As our study was conducted in EDs of university hospitals that were tertiary hospitals, patients who had GF needing urgent care to get relief of severe pain visited primary hospitals that were more convenient than visiting EDs of university hospitals. Besides that, duration periods for study, inclusion criteria, and geographic area were also different.

GF was a cause of acute arthritis that presented in EDs [7]. For GF, arthrocentesis was seldom performed [7, 11, 14, 34], especially in EDs [7, 10]. Likewise in this study, arthrocentesis (29.3%) was seldom performed in the EDs. Underlying gout and tophi presentation might be reasons for this result. Moreover, the first metatarsopharyngeal joints that were classic in gouty arthritis and also were small joints difficult to aspirate for physicians who are not rheumatologists, giving another possible reason for seldom performing arthrocentesis at EDs.

Despite the variation of SUA level found in GF [35] and minimal benefit for acute management, an evaluation of SUA in EDs was still performed in our study. Inappropriate gout knowledge and no mention about this item in 2012 TRA-GMG were possible causes of our result.

The evaluation of management of GF, especially inpatient department, was presented in previous studies [11, 14, 34]. In our current study that focused on EDs, investigations with arthrocentesis were lower but the visits having a follow-up appointment were higher than in previous studies [11, 14, 34]. The situation in EDs including high workloads and unskilled arthrocentesis may affect the different management results between EDs and admitted patients. Since arthrocentesis was rarely performed in EDs, the definite diagnosis of GF may be in doubt, and the medications were already prescribed by non-rheumatologists to stop severe pain. For the safety of patients after being discharged from EDs, the proportion of a follow-up appointment from EDs was higher than inpatient departments. Therefore, these pitfalls were different between EDs and in admitted patients.

Many studies demonstrated that the management of GF, including investigation and treatment were inappropriate and suboptimal [10, 11, 14, 28, 34]. However, only a few studies reported the management of GF in EDs [10, 28] where suboptimal management was also presented [10]. In our study, there was also suboptimal management; however the proportion of suboptimal management was less than a previous study [10] in EDs and HM.

The adjustment of medications to reduce inflammation in GF depends on the duration of the GF and the safety of patients, especially for those with underling disease [3]. The 2012 TRA-GMG recommended only monotherapy, including colchicine, NSAIDs, or systemic corticosteroid treatment to get rid of inflammation in GF. Accordingly, monotherapy was the first recommendation for acute GF in the 2012 ACR guidelines for the management of gout [3]; monotherapy was also the most common in this study.

Although the 2012 TRA-GMG did not make recommendations about combination therapy, the 2012 ACR guidelines for the management of gout were also published in the period of our study, and combination therapy was mentioned in the guidelines [3]. Therefore, the guidelines might influence decisions of clinicians. This was our hypothesis for finding combination therapy included in EDs and HM in our study. However, combination therapy other than that recommended in the 2012 ACR guidelines [3], including NSAIDs plus corticosteroids and triple regimen (NSAIDs and colchicine plus corticosteroids) was also found in our study. This result reflected overuse and inappropriate medications for treatment of GF.

Although visits that had no medications prescribed were found in our study, we found that these visits had a history of gout, colchicine, or education. Also, we expected that they previously had other medications from private clinics or hospitals. Therefore, internists or general clinicians just advised for an adjusted dose of the medications for GF.

Particularly for gout, inappropriate medication, especially NSAIDs, was prescribed in EDs and for HM [27]. In our study, about one-third of the patients used NSAIDs although they had not been evaluated for renal function, and NSAIDs were used in visits with severe liver disease. A lot of patients who needed health services went to EDs; this high work load caused problems for clinicians in EDs [36]. A lack of evaluation about necessary investigations might be a result of this situation. In addition, most clinicians who worked in EDs were internists or in general practice. Therefore, these were factors including the knowledge of clinicians, and the environment in EDs that might have resulted in suboptimal management and investigation and the pitfalls including *over* or *under investigation*, *over* or *under treatment* and *overuse medication* in our results.

Additionally, although national guidelines were published, suboptimal management, including long term and acute GF management were inadequately presented [8, 37, 38]. Factors, such as perceptions of illness by clinicians and unawareness of the published gout treatment recommendations play a vital role in these problems [18, 19]. Moreover, the promotion of clinical guidelines through printed material was minimally effective for clinicians [39, 40]. The results of our study showed suboptimal management of GF in EDs based on the 2012 TRA-GMG. This reflected the failure of guidelines or recommendations to be used in clinical practice, particularly in EDs.

Whereas there has been no study that demonstrates a strategy for improved management of GF in EDs, the use of “wall-posters, educational sessions, and intranet web-link to a hospital-wide protocol” could significantly improve management of GF in hospitalization particularly for non-rheumatologists and has been proposed [41]. We assumed that the strategy may be applied to improve the quality of management of GF in EDs since the pattern of this strategy would resolve many barriers between clinicians and guidelines [16–19] at EDs.

The present study is the first study in Thailand to report the prevalence of GF in EDs while evaluating the quality of gout care in EDs based on the national guideline for gout management. A major strength of our study was a reflection for real practice about management of GF in EDs as our study used a retrospective study design. Therefore, clinicians in EDs were unaware of evaluation of their management when they were practicing. In addition, this study used the 2012 TRA-GMG for standards about management. Therefore, our study had certain standard items taken from the 2012 TRA-GMG for evaluation.

Recognizing these vital strengths, there were still several limitations to our study. Since this study was a retrospective study, offering a benefit to our study (as mentioned above), some data had not been recorded. This was limited by the design. This study reviewed only primary diagnosis by ICD-10 in gout at EDs and the visits that were only diagnosed by ICD-10, but no definite diagnosis by arthrocentesis or the classification criteria, was included. Therefore, prevalence might be under evaluated and reflected only primary diagnosis by ICD-10 in gout at EDs, respectively. Other than that, this study only collected data from medical schools that reflected tertiary hospitals, the results might not reflect all of the EDs in Thailand. Additionally, as there was no significant statistical cut-point of the number or proportion for demonstration of appropriate management that compared with a standard, it was difficult to conclude which items had a statistically significant appropriate management in comparison with the standard guideline in our study. Many studies [8, 10–12, 14] including our study were only reported in proportion. This limitation ought to be studied in the future.

Furthermore, the characteristic data of clinicians, including duration or experience of clinical practice and subspecialty and factors that affected clinical adjustment or management of GF in EDs were not evaluated; therefore, differences in clinicians and these factors may have affected the quality of gout care. Our study was a retrospective design and only focused on emergency management of GF that had different managements from other stages of gout [3–5] but did not include previous and postoperative visits to the outpatients departments; the cohort study design would be preferred in the future. Finally, quality of gout care in our study was based on the 2012 TRA-GMG; clinicians who treated GF patients in EDs were not surveyed about their familiarity and agreement with this publication. Therefore, these limitations should be considered in interpreting the results of our study. The characteristics of clinicians who worked in EDs or the factors that affected clinical adjustment or management in EDs and were surveyed about their acceptance of the 2012 TRA-GMG should be included in further research. Finally, besides guidelines or recommendations, other strategies should to be considered to improve the quality of gout care, especially in EDs.

## Conclusions

In summary, our study also demonstrated that quality of gout care in EDs was not good and inappropriate, particularly with regard to investigations and pharmacological management based on the 2012 TRA-GMG. Gaps between clinicians and guidelines, the knowledge of clinicians, and overcrowding in EDs were hypothesized in the results.

## Supplementary information

**Additional file 1: Figure S1.** The flow of visits in the study.

## Data Availability

Not applicable

## Abbreviations

GF: Gout flare
EDs: Emergency departments
TRA-GMG: Thai Rheumatism Association Guidelines for Management of Gout
ICD-10: International Classification of Diseases, the tenth revision
HM: Home medications
MSU: Monosodium urate crystals
ACR: American College of Rheumatology
EULAR: European League Against Rheumatism
NSAIDs: Non-steroidal anti-inflammatory drugs
COX: Cyclooxygenase
PPI: Proton pump inhibitor
SUA: Serum uric acid
eGFR: Estimated glomerular filtration rate
ULT: Urate lowering therapy
