# Supplementary material for: Quality of gout care in the emergency departments: a multicentre study
Source: BMC Emerg Med. 2020 Apr 20;20:27. doi: 10.1186/s12873-020-00319-w (PMC7171834; doi:10.1186/s12873-020-00319-w)
Supplement: Supplementary file 1 — Additional file 1: Figure S1. The flow of visits in the study. [file 12873_2020_319_MOESM1_ESM.docx]

**Figure 1** The flow of visits in the study.

Excluded (n=1,619,495)

- Not meeting inclusion criteria

(n=1,619,495)

Excluded (n=1,264)

- The primary diagnosis of gout by ICD-10 but no a clinical presentation of acute arthritis in this visits (n=1,102)

- Missing OPD card (n=162)

Analyzed (n=632)

- Excluded from analysis (n=0)

Excluded (n=12)

- Data missing of medications in emergency departments or home medications (n=12)

Gout flare was defined as a clinical presentation of acute arthritis in visits that had been identified by the primary diagnosis of gout (n=644)

The primary diagnosis of gout using the International Classification of Diseases, the tenth revision (ICD-10) code, including M10.0, M10.1, M10.2, M10.3, M10.4 and M10.9 at emergency departments (n=1,908)

Total visits in emergency departments from

1 January 2012 to 31 December 2016 (n=1,621,403)
